# The RP-Mdm2-p53 Pathway and Tumorigenesis

**DOI:** 10.18632/oncotarget.228

**Published:** 2011-03-04

**Authors:** Paula L. Miliani de Marval, Yanping Zhang

**Affiliations:** ^1^ Department of Radiation Oncology, School of Medicine, the University of North Carolina at Chapel Hill, Chapel Hill, NC; ^2^ Lineberger Comprehensive Cancer Center, School of Medicine, the University of North Carolina at Chapel Hill, Chapel Hill, NC; ^3^ Department of Pharmacology, School of Medicine, the University of North Carolina at Chapel Hill, Chapel Hill, NC

**Keywords:** Mdm2, p53, cancer, nucleolus, ribosome biogenesis, RPL11

## Abstract

The dynamic processes of cell growth and division are under constant surveillance. As one of the primary “gatekeepers” of the cell, the p53 tumor suppressor plays a major role in sensing and responding to a variety of stressors to maintain cellular homeostasis. Recent studies have shown that inhibition of ribosomal biogenesis can activate p53 through ribosomal protein (RP)-mediated suppression of Mdm2 E3 ligase activity. Mutations in Mdm2 that disrupt RP binding have been detected in human cancers; however, the physiological significance of the RP-Mdm2 interaction is not completely understood. We generated mice carrying a single cysteine-to-phenylalanine substitution in the central zinc finger of Mdm2 (Mdm2^C305F^) that disrupts Mdm2’s binding to RPL11 and RPL5. Despite being developmentally normal and maintaining an intact p53 response to DNA damage, the Mdm2^C305F^ mice demonstrate a diminished p53 response to perturbations in ribosomal biogenesis, providing the first *in vivo* evidence for an RP-Mdm2-p53 signaling pathway. Here we review some recent studies about RP-Mdm2-p53 signaling and speculate on the relevance of this pathway to human cancer.

## INTRODUCTION

p53 is a tumor suppressor that has been implicated in regulating an assortment of cellular events including cell cycle arrest, apoptosis, senescence, and aging. p53 activation and function depend heavily on post-translational modifications, mostly triggered by a variety of cellular stresses. Depending on the nature of the stress signal, p53 activates genes such as p21, 14-3-3 sigma, GADD45, and PAI1 to promote cell survival or senescence [1-3]. The activation of these target genes contributes ultimately to p53’s tumor suppression function. A recently revealed aspect of p53 function is its ability to regulate cellular metabolism. Through the activation of genes such as TIGAR, SCO2 and SESTRINS, p53 has been found to modulate mitochondrial function and cellular metabolism [4]. In addition, p53 has been found to interact with cytoplasmic ribosomes and is involved in translational control and ribosome biogenesis by influencing ribosomal RNA transcription factors and RNA Pol-I (RNA Polymerase-I) [5]. A recent report provides yet another insight into the already intricate network of p53 function and regulation. Mdm2, a master regulator of p53, has been found to modulate the p53 ribosomal stress response through its interaction with ribosomal proteins (RP) during periods of decreased and increased ribosomal biogenesis [6].

Mdm2 is an E3 ubiquitin ligase to promote p53 ubiquitination, leading to nuclear export of p53 and degradation by the proteasome. Mdm2 ablation in mice results in early embryonic lethality due to elevated levels of p53-induced apoptosis, and this phenotype is reversed by the simultaneous deletion of p53, demonstrating the importance of Mdm2’s role in suppressing p53 [7-8]. Mdm2 protein has three well-defined regions: the N-terminus for p53 binding followed by a central region that contains acidic and zinc finger domains and a C-terminal RING finger domain. The RING finger region possesses the ubiquitin ligase activity as well as a binding site for the Mdm2 homologue MdmX [9]. While Mdm2 is well recognized to negatively regulate p53 through its N-terminal and C-terminal domains, many other proteins can interact with Mdm2’s central domain in order to modulate its function. Most of these proteins associate with Mdm2 as a result of acute stimuli; among these are p14^ARF^/19^ARF^ and the ribosomal proteins RPL5, RPL11, RPL23, RPS7, RPL26 and RPS3.

## REGULATION OF RIBOSOMAL BIOGENESIS AND CELL CYCLE CHECKPOINT

The regulation of ribosome biogenesis is fundamental for cell growth control. It involves the coordination of multiple steps including synthesis and processing of ribosomal RNA (rRNA), synthesis of ribosomal proteins and their import into the nucleus, assembly of ribosome subunits, transport of the mature 40S and 60S subunits to the cytoplasm, and assembly of 80S ribosome in the cytoplasm. The ribosomal subunits subsequently bind to and translate mRNAs into polypeptides. In a vigorously growing cell, ribosome biogenesis is a major consumer of cellular energy and resource. Thus, as growth conditions change, cells must rapidly rebalance ribosome production with the availability of resources [10]. Ribosome biogenesis requires the transcription of rRNA by RNA pol I [11-12] and coordinated synthesis of ribosomal proteins. Impaired expression of ribosomal proteins has been associated with various functional consequences including tumorigenesis as well as growth abnormalities in humans and mice [13-15]. Following the discovery of binding between RPL11 (L11) and Mdm2 [16-17], the observations of Bhat et al. [18] provided insights into the physiological role of the L11-Mdm2-p53 pathway by characterizing the nature of this association under growth inhibitory conditions triggered by serum starvation. It has been shown that serum starvation activates p53 and induces cell cycle arrest in an L11-dependent manner through a mechanism involving translocation of L11 from the nucleolus to the nucleoplasm. The model perceived from a myriad of *in vitro* data suggested that when cells sense nutrient-shortage stress, ribosomal proteins are released from the nucleolus to the nucleoplasm where they can bind to Mdm2 and inhibit its E3 ligase activity, leading to activation of p53.

## ALTERATIONS IN THE RP-MDM2-P53 PATHWAY AND TUMORIGENESIS

The relevance of these findings was further substantiated by the discovery of Mdm2 mutations in the region that binds to L11 in human cancers [19-20]. It was demonstrated that these cancer-associated mutations are confined to the central zinc finger of Mdm2 and specifically disrupt the interaction between Mdm2 and L11 or L5 *in vitro* [21]. As a result, the Mdm2 mutants (e.g. Mdm2^C305F^) are refractory to inhibition by ribosomal proteins and are maintained in a p53-suppressive mode. *in vitro*, it was demonstrated that the integrity of the zinc finger is essential for Mdm2 to efficiently induce degradation of p53 and itself, but it was not strictly required for ubiquitination of either one [18]. These data uncovered a novel mechanism for Mdm2 oncogenic activation and a new level of Mdm2-p53 regulation, and together with previous studies, suggest that three distinct modes of p53 regulation exist: phosphorylation of Mdm2 and p53 by various kinases induced by DNA damage, induction of p14^ARF^-/p19^ARF^-Mdm2 interaction triggered by oncogenic insults, and the induction of RP-Mdm2 interaction triggered by signaling malfunctions in ribosomal biogenesis. Although it was clear, based on the above studies, that the RP-Mdm2-p53 pathway conveyed signals sensing nutrient status to ribosomal biogenesis and cell cycle control, it was crucial to unravel the physiological consequences of such interactions. Macias et al [6] took those findings to a physiological level by providing *in vivo* evidence for a prevailing p53 checkpoint mediated by RP-Mdm2 interaction. The study created a genetic mouse model to elucidate the *in vivo* physiological relevance of the interaction between L11 and Mdm2, and determined how impaired p53 function affects cell growth and tumor development. This knockin mouse carries a cysteine-to-phenylalanine mutation (Mdm2^C305F^) in the zinc finger region, preventing Mdm2-L11 binding. Under normal conditions, Mdm2^C305F/C305F^ homozygous mutant mice showed no obvious phenotypical or developmental differences compared to their wild type counterparts, nor did they display a genetic predisposition to the development of spontaneous tumors. Mouse embryonic fibroblast (MEF) cells homozygous for the Mdm2^C305F^ mutation displayed a normal DNA damage response to various DNA-damaging reagents including Doxorubicin, UV, γ-irradiation, as well as a high dose of Actinomycin D. Whole body γ-irradiation yielded indistinguishable levels of p53 target gene activation and apoptosis between wild type and Mdm2^C305F^ mutant groups. In contrast, treatment with reagents that specifically targeted ribosomal biogenesis confirmed that impaired L11-Mdm2 binding results in an attenuated p53 response, thus failing to induce cell cycle arrest in the Mdm2^C305F^ mutant mice. Altogether, it was established with this *in vivo* model that the RP-Mdm2 interaction transduces cellular stress signals from ribosomal biogenesis specifically, but not DNA damage, to p53. However, the role of the RP-Mdm2-p53 pathway in tumorigenesis had yet to be determined. To address this question, the oncoprotein c-Myc was studied in conjunction with the Mdm2^C305F^ mutation. c-Myc is overexpressed in numerous types of cancers and is known to regulate ribosomal biogenesis through direct transactivation of many genes involved in ribosomal biogenesis. In particular, L11 is a direct transcriptional target of c-Myc and interferes with c-Myc-dependent transactivation by competing with its cofactors for DNA binding [22]. L11 also seems to affect c-Myc expression levels, as knockdown of L11 increases the mRNA and protein levels of endogenous c-Myc [22]. Thus, the coordinated regulation of L11 and c-Myc may function in a negative feedback loop, acting as a sensor of abnormal ribosome biogenesis and consequently limiting c-Myc-driven proliferation [23]. To explore whether the L11-Mdm2-p53 pathway collaborates with c-Myc-induced tumorigenesis, or whether these two pathways function independently, Macias et al. developed compound mice by breeding Eμ-Myc transgenic mice into the *Mdm2^C305F/C305F^* background. *Eμ-Myc/Mdm2^C305F/C305F^* compound mice displayed an accelerated death rate due to an early onset of lymphomas. Furthermore, it was clearly shown that in organs derived from non-tumor-bearing Eμ-*Myc/Mdm2^C305F/C305F^* compound mice there was an increased level of expression of L11 and L5 compared to non-Eμ-*Myc* control mice. Cellular fractionation experiments using splenocytes derived from these mice showed that c-Myc-induced L11 saturated both the nucleolar and nucleoplasmic fractions. Co-immunoprecipitaion experiments from splenic extracts showed that L11 derived from Mdm2^C305F^ mice was unable to associate with the mutant Mdm2 and, in turn, the p53 response to c-Myc-induced tumorigenesis was impaired in these mice [22]. These investigations demonstrate that the integrity of the RP-Mdm2-p53 pathway is crucial for protection against c-Myc-induced lymphomagenesis *in vivo*.

p14^ARF^/p19^ARF^ acts as a tumor suppressor in the p53 pathway. It binds to Mdm2 and blocks its E3 ligase activity, leading to concomitant stabilization and accumulation of p53. The functional consequence of p19^ARF^’s regulation of p53 through inhibition of Mdm2 is evidenced by the ability of ectopically expressed p19^ARF^ to restore a p53-imposed G1 cell cycle arrest that is otherwise abrogated by Mdm2 [24]. Interestingly, p19^ARF^ is required for p53 activation from proliferative signals such as c-Myc activation, but not for activation in response to ultraviolet or ionizing radiation [25].

**Figure 1:**
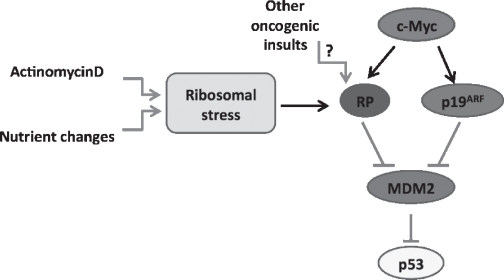
Mdm2 conveys stress signals to p53 through multiple pathways Changes in nutrient availability or treatment with low doses of Actinomycin D lead to nucleolar (ribosomal) stress. As a result, ribosomal proteins L5 and L11 bind to and inhibit the E3 ligase function of Mdm2, resulting in p53 stabilization and activation. In addition, oncogenic insults, such as aberrant expression of c-Myc, induce the expression of both p19^ARF^ and ribosomal proteins. The RP-MDM2-p53 and p19^ARF^-Mdm2-p53 represent two parallel signaling pathways in response to oncogenic c-Myc stimulation.

Considering the intimate connections between c-Myc, p19^ARF^, Mdm2, RP and p53, it is logical to question how these pathways communicate or cooperate in the context of tumorigenesis. Are the RP-Mdm2-p53 and p19^ARF^-Mdm2-p53 pathways functioning in a linear or a parallel fashion? Using mouse embryo fibroblast (MEF) cells derived from wild type, p19^ARF^-null, and Mdm2^C305F/C305F^ mice, it was demonstrated that the p19^ARF^-Mdm2-p53 pathway acts independently from the RP-Mdm2-p53 pathway in terms of c-Myc’s induction of p53. Two independent approaches helped to arrive at this conclusion. First, transient expression of c-Myc resulted in a partial p53 increase in both p19^ARF^-null and Mdm2^C305F/C305F^ MEFs, indicating that each pathway contributes separately to c-Myc’s activation of p53. Second, ribosomal stress induced by a low concentration of Actinomycin D in p19^ARF^-null and wild type MEFs, but not Mdm2^C305F/C305F^ MEFs, led to elevated levels of both Mdm2 and p53 as well as increased binding between Mdm2 and L11, indicating that p19^ARF^ is dispensable for induction of p53 by low-level Actinomycin D, which is known to activate p53 through the RP-Mdm2 pathway. Moreover, both Eμ-*Myc/Mdm2*^+/+^ and Eμ-*Myc/Mdm2*^C305F/C305F^ mice showed induced levels of p19^ARF^ in tumor-bearing mice compared to non-tumor-bearing littermates, suggesting that p19^ARF^-Mdm2-p53 signaling remains intact in the *Mdm2*^C305F/C305F^ mice. In summary, these studies established that p19^ARF^ is likely not required for ribosomal stress-induced p53 activation and that the RP-Mdm2-p53 pathway is an important barrier for defending against oncogenic c-Myc-induced tumorigenesis, independent from p19^ARF^-Mdm2-p53 signaling (Figure 1).

Through its interaction with Mdm2, p19^ARF^ mediates a p53-dependent checkpoint in response to a broad range of oncogenic insults including elevated expression of c-Myc, E2F1, Ras, E1A, and v-Abl [26]. In this sense, it is conceivable that the L11-Mdm2-p53 signaling also mediates a p53-dependent checkpoint in response to deregulated oncogenes that promote superfluous ribosome biogenesis. For instance, E2F1 specifically interacts with and enhances rRNA promoter activity through two E2F1-binding sequences [27], and oncogenic Ras influences mTOR activity, and thus protein translation, by modulating the PI3K-Akt signaling cascade [26]. In both cases, the oncogenic activity of E2F1 and Ras promotes ribosomal biogenesis either directly or indirectly. Whether the L11-Mdm2-p53 signaling pathway is specific to c-Myc, or whether it is a general oncogenic surveillance pathway that responds to a broad range of deregulated oncogenes, including E2F1 and Ras, remains to be determined. Several reports have described upregulation of the ribosomal biogenesis pathway in mice subjected to a two-stage carcinogenesis protocol [28-29]. Treatment of mouse skin with the genotoxic agent 7,12-Dimethylbenz(a)anthracene (DMBA) creates Ras mutations, combined with serial applications of the tumor promoter 12-O-tetradecanoylphorbol-13-acetate (TPA) [30], lead to the development of mouse skin papillomas. With sufficient genomic instability, these treatments can also lead to malignant progression and development of squamous cell carcinomas [31-32]. Thus, using skin as a model, it would be valuable to test the possibility that Ras activation collaborates with the RP-Mdm2-p53 pathway to induce malignant progression and carcinogenesis. In addition, both Rb family proteins and p53 have been reported to negatively regulate Pol-I-dependent rRNA transcription (reviewed by Ruggero and Pandolfi [15]). Can the L11-Mdm2-p53 pathway and the Rb/p53-Pol-I pathway intertwine in a feedback loop to positively regulate each other? Studying these issues could aid in our understanding of how cell growth control is coordinated with cell cycle regulation.

It has recently been reported that p53 regulates mitochondrial respiration [33]. A direct transcriptional target of p53, SCO2, was identified that modulates aerobic respiration. Deregulation of SCO2 more or less recapitulates the Warburg Effect, a phenomenon in which cancer cells rely primarily on glycolysis rather than oxidative phosphorylation to generate energy even in the presence of sufficient oxygen. Interestingly, the study establishes a genetic pathway in which the inactivation of p53 may promote tumorigenesis by decreasing cellular dependence on oxygen, potentially permitting growth in more hypoxic environments. Thus, it is possible that by taking advantage of the Mdm2^C305F^ animal model, we could evaluate the functional consequences of Mdm2 mutation under ribosomal stress conditions. Evaluating the outcome of changes in nutrient availability through food deprivation, calorie restriction and exercise, and the impact on mitochondrial response under those metabolic changes, would allow us to better understand the non-tumor suppressor, pro-survival functions of p53 as well as to explain why this gene is so widely conserved during evolution
